# Population Dynamics of Perception and Emergence of Translational Membranes

**DOI:** 10.1007/s42113-025-00241-z

**Published:** 2025-04-07

**Authors:** James P. Crutchfield, Steve T. Piantadosi

**Affiliations:** Complexity Sciences Center and Department of Physics and Astronomy, University of California at Davis, One Shields Avenue, Davis, CA 95616 USA

**Keywords:** Population dynamics, Structural complexity, Spatial dimension, Autocatalytic network

## Abstract

**Abstract:**

At root, perception converts external information from an environment to signals of use in downstream cognition. We explore a population of interacting perceptual agents whose adaptive internal structure transforms input information to outputs. We show that autocatalytic networks of structural transformations—represented by $\epsilon$-transducers—spontaneously emerge. Moreover, their population dynamics—who flourishes and with whom they interact—differ substantially between spatial (geographically distributed) and nonspatial (panmixia) populations. Generally, regions of spacetime-invariant autocatalytic networks—or *domains*—emerge in geographically distributed populations. These are separated by *functional membranes* of complementary $\epsilon$-transducers that actively translate between the domains and are responsible for their growth and stability. We analyze both spatial and nonspatial populations, determining the algebraic properties of the autocatalytic networks that allow for space to affect the dynamics and so generate autocatalytic domains and membranes. In addition, we analyze populations of intermediate spatial architecture, delineating the thresholds at which spatial memory (information storage) begins to determine the character of the emergent autocatalytic organization.

**Author Summary:**

How did perception emerge in early evolution? The first biological replicators are believed to have been autocatalytic networks of functional molecules that collectively were capable of self-reproduction. In a purely replicative system, though, how could a mechanism spontaneously arise that selects for individuals with different structural properties and transformational capabilities? We answer this question by demonstrating how selection emerges and how the spatial dimension of a population directly affects the nature of successful individuals and their autocatalytic networks. We provide a detailed mathematical model that includes both the spatial population dynamics and measures of structural organization for populations, delineating the algebraic characteristics required for cooperative survival. The results indicate how evolutionary processes can harness the dimension of space to support varying kinds of structural perception and functional collective organization.

## Introduction

When stripped of its physical, chemical, and biological substrates, perception at its most fundamental, operational level is information transduction. Perception is not an isolated process, though. Rather it is most prominent in agents that survive in diverse, structured environments. What happens when a group of information transducers (cognizing agents) interact, providing an environment for each other?

In the following, we have in mind biological perception seen in bacterial populations (Marzen & Crutchfield, 2018) and molecular sensors (Marzen & Crutchfield, 2025). It analyzes one of the simplest models of replicating cognitive agents, showing that the population spontaneously organizes and, in the process, changes how the agents perceive, store, and transform structured information from their adapting environment. In its simplicity, the basic model is general enough to apply quite broadly.

Almost certainly, the sophisticated mechanisms for self-replication found today in living cells were not present in the earliest replicators (Samáry & Demeter, 1987; Maynard Smith & Szathmary, 1995; Castilian et al., 1999). Instead, some hypothesized that the first replicators were autocatalytic networks of functional molecules that collectively were capable of self-reproduction (Eigen, 1971; Eigen & Schuster, 1977). Numerous studies have been devoted to these and analogous networks with the hope of understanding pre-biotic evolution (Ray, 1991; Adami & Brown, 1994; Rasmussen et al., 1992). A particular class of such models represented network elements with mathematical constructs, including regular expressions, the $\lambda$-calculus (Fontana, 1991), and, recently, $\epsilon$-machines (Crutchfield & Gornerup, 2006; Gornerup & Crutchfield, 2008). $\epsilon$-Machines are especially useful since they support well defined and computable measures of structural complexity (Ellison et al., 2009) and, equally important, these measures extend directly to networks of interacting $\epsilon$-machines and so to hierarchical complexity.

Early models often assumed a *Turing gas* in which every network element has an equal chance of interacting with every other, thereby ignoring a network’s spatial configuration. However, natural and engineered evolutionary systems are not architected this way since elements of physical systems always have some spatial relationship that determines which components interact. This observation led to studies of spatial pattern formation in evolutionary and autocatalytic systems (Kirner et al., 2000, 2002; Ackermann et al., 2006).

Here, we consider networks of single-state $\epsilon$-machines—a simplifying initial focus that helps to highlight the role of population architecture. We show that the behavior of spatially distributed populations differs substantially from that of the nonspatial Turing gas. More to the point, we determine the algebraic properties of the network that lead to the emergence of distinctive organizations.

## Methods

### Background

$\epsilon$*-Machines* serve as models of stochastic finite and infinite computation (Crutchfield & Young, 1989; Crutchfield, 1994; Crutchfield & Shalizi, 1999). An $\epsilon$-machine consists of a set $\boldsymbol{ \mathcal {S} }$ of *causal states* and a set of *transitions* between those states. In the particular variation used here ($\epsilon$*-transducers* Barnett & Crutchfield, 2015), each transition is labeled by both an input symbol $x \in \mathcal {A}$ and an output symbol $y \in \mathcal {A}$. An $\epsilon$-machine in state $\sigma \in \boldsymbol{ \mathcal {S} }$ reads an input symbol *x* and chooses from all transitions from $\sigma$ the one labeled *x*. The $\epsilon$-machine follows the chosen transition to a next state $\sigma ^\prime \in \boldsymbol{ \mathcal {S} }$ while emitting the output symbol *y* corresponding to that transition. In this way, $\epsilon$-machines can be viewed as mapping an input language to an output language, perhaps probabilistically.

Following Crutchfield and Gornerup (2006), and Gornerup and Crutchfield (2008) we focus on single-state $\epsilon$-machines over the input–output alphabet $\mathcal {A}= \lbrace 0, 1 \rbrace$. (Results for multi-state $\epsilon$-machines appear in a sequel. Our goal here is to highlight the effects of space, uncomplicated by the richness that comes with using multi-state $\epsilon$-machines.) There are 16 such $\epsilon$-machines and we denote the set of all them by $\mathbb {T}$. Each $\epsilon$-machine can be represented as a $2 \times 2$ binary matrix *M*, where $M_{ij} = 1$ means that the $\epsilon$-machine reads in symbol $i-1$ while emitting symbol $j-1$. We number the 16 $\epsilon$-machines—$\mathbb {T}\equiv \{ T_0, \ldots , T_{15} \}$—by finding the decimal equivalent of the binary number $M_{11} M_{12} M_{21} M_{22}$ for each $\epsilon$-machine. Thus, for example, $\epsilon$-machine $T_{11}$ has the matrix representation:

1 $$\begin{aligned} M = \left[ \begin{array}{cc} 1& 0\\ 1& 1 \end{array} \right] ~. \end{aligned}$$

$\epsilon$-Machine pairs interact in a population by composition, under which $\mathbb {T}$ is closed and forms a monoid (Holcombe, 1982). With the matrix representation, $\epsilon$-machine composition—$T_b \circ T_a$—is simply matrix multiplication where, after multiplying, any positive matrix element is set to 1. From this, it is straightforward to compute the *interaction matrix* $\mathbb {M}$, where $T_a \circ T_b = T_c$ if and only if $\mathbb {M}_{a+1, b+1} = c$, where $a,b,c \in \{0, \ldots , 15\}$:

2 $$\begin{aligned} \mathbb {M}= \left[ {\begin{smallmatrix} 0& 0& 0& 0& 0& 0& 0& 0& 0& 0& 0& 0& 0& 0& 0& 0\\ 0& 1& 2& 3& 0& 1& 2& 3& 0& 1& 2& 3& 0& 1& 2& 3\\ 0& 0& 0& 0& 1& 1& 1& 1& 2& 2& 2& 2& 3& 3& 3& 3\\ 0& 1& 2& 3& 1& 1& 3& 3& 2& 3& 2& 3& 3& 3& 3& 3\\ 0& 4& 8& 12& 0& 4& 8& 12& 0& 4& 8& 12& 0& 4& 8& 12\\ 0& 5& 10& 15& 0& 5& 10& 15& 0& 5& 10& 15& 0& 5& 10& 15\\ 0& 4& 8& 12& 1& 5& 9& 13& 2& 6& 10& 14& 3& 7& 11& 15\\ 0& 5& 10& 15& 1& 5& 11& 15& 2& 7& 10& 15& 3& 7& 11& 15\\ 0& 0& 0& 0& 4& 4& 4& 4& 8& 8& 8& 8& 12& 12& 12& 12\\ 0& 1& 2& 3& 4& 5& 6& 7& 8& 9& 10& 11& 12& 13& 14& 15\\ 0& 0& 0& 0& 5& 5& 5& 5& 10& 10& 10& 10& 15& 15& 15& 15\\ 0& 1& 2& 3& 5& 5& 7& 7& 10& 11& 10& 11& 15& 15& 15& 15\\ 0& 4& 8& 12& 4& 4& 12& 12& 8& 12& 8& 12& 12& 12& 12& 12\\ 0& 5& 10& 15& 4& 5& 14& 15& 8& 13& 10& 15& 12& 13& 14& 15\\ 0& 4& 8& 12& 5& 5& 13& 13& 10& 14& 10& 14& 15& 15& 15& 15\\ 0& 5& 10& 15& 5& 5& 15& 15& 10& 15& 10& 15& 15& 15& 15& 15\\ \end{smallmatrix}} \right] ~. \end{aligned}$$

### $\epsilon$-Machine Soups

A population, or simply a *soup*, $\Gamma$ is a configuration of an $n \times n$ regular toroidal lattice. At each time $t=0,1,2,\ldots$, every lattice location (*i*, *j*) contains a single $\epsilon$-machine, denoted $\Gamma _{i,j}^t \in \mathbb {T}$. The population size is $N = n^2$. Each location (*i*, *j*) is initialized to contain an $\epsilon$-machine uniformly chosen at random from $\mathbb {T}$.

The population dynamics are specified by $\epsilon$-machine composition, interaction, and update. We define a function $\theta : \mathbb {T}\times \mathbb {T}\times \mathbb {T}\rightarrow \mathbb {T}$ by:

3 $$\begin{aligned} \theta (T_{a}, T_{b}, T_{c}) = {\left\{ \begin{array}{ll} T_{a} \circ T_{c} & \text { if } T_{a} \circ T_{c} \ne T_{0} \\ T_{b} & \text { otherwise } \end{array}\right. } ~. \end{aligned}$$

We write $\theta (A, B, C)$ for sets $A,B,C \subseteq \mathbb {T}$ as shorthand for the set $\lbrace \theta (a,b,c): a \in A, b \in B, c \in C \rbrace$. We must use $\theta$ to prevent the non-$\epsilon$-machine $T_0$ from being produced since the fact that $T_0 \circ T_a = T_a \circ T_0 = T_0$ for all $T_a\in \mathbb {T}$ implies that if $T_0$ could be produced, it comes to dominate any population.

Each time step *t*, we choose a location (*i*, *j*) at random and set:

4 $$\begin{aligned} \Gamma _{i,j}^{t+1} = {\left\{ \begin{array}{ll} \theta \left( \Gamma _{i-1,j}^{t}, \Gamma _{i,j}^t, \Gamma _{i+1,j}^{t} \right) & \!\! \text {with probability } \tfrac{1}{4}\\ \theta \left( \Gamma _{i+1,j}^{t}, \Gamma _{i,j}^t, \Gamma _{i-1,j}^{t} \right) & \!\! \text {with probability } \tfrac{1}{4}\\ \theta \left( \Gamma _{i,j-1}^{t}, \Gamma _{i,j}^t, \Gamma _{i,j+1}^{t} \right) & \!\! \text {with probability } \tfrac{1}{4}\\ \theta \left( \Gamma _{i,j+1}^{t}, \Gamma _{i,j}^t, \Gamma _{i,j-1}^{t} \right) & \!\! \text {with probability } \tfrac{1}{4}\\ \end{array}\right. } \end{aligned}$$

and set $\Gamma _{k,l}^{t+1}=\Gamma _{k,l}^t$ for all $(k,l) \ne (i,j)$. Thus, two vertical or horizontal neighbors to $\Gamma _{i,j}$ are chosen, composed, and the $\epsilon$-machine resulting from their composition is used to replace $\Gamma _{i,j}^t$, if it is not $T_0$. This replacement scheme is meant to be locally analogous to the replacement scheme used in Crutchfield and Gornerup (2006), and Gornerup and Crutchfield (2008), which will facilitate direct comparisons in the following.

In addition, at each time step a certain amount of diffusion of $\epsilon$-machines occurs due to spatial mixing. For this, let $\zeta _v$ be a Gaussian distribution with variance *v* and mean 0. At each time step, *c* $\epsilon$-machines are chosen at random in $\Gamma$ and each is swapped with a random $\epsilon$-machine at a distance chosen from the distribution $\zeta _v$. For more generality, we allow *c* to be any real number and swap *c* $\epsilon$-machines per time step on average.

When *v* and *c* are large, there is considerable spatial mixing and one expects the dynamics to behave like a nonspatial population, where $\epsilon$-machines have an equal chance of interacting with every other. However, when *v* and *c* are small, there is little spatial mixing and, as we shall see, the population dynamics change substantially.

To summarize, as a stochastic dynamical system the soup’s state at time *t* is the population’s *configuration* $\Gamma ^t$. While Eq. 4 determines the local probabilistic update at each site, we use $\Theta$ to formally denote the global (probabilistic) update for the entire configuration over one time step:

5 $$\begin{aligned} \Gamma ^{t+1} = \Theta \circ \Gamma ^t ~. \end{aligned}$$

And so, one goal is to understand the trajectories $\{\Gamma ^0, \Gamma ^1, \ldots \}$. Another is to analyze the structure inside the $\Gamma ^t$. For this, we use $\sigma _{\widehat{n}}$ to denote a spatial shift of the soup configuration:

6 $$\begin{aligned} \Gamma ^\prime = \sigma _{\widehat{n}} \circ \Gamma ~, \end{aligned}$$

where $\widehat{n} = (\Delta i, \Delta j)$ is the vector by which the configuration is shifted horizontally and vertically:

7 $$\begin{aligned} \Gamma _{k,l}^\prime&= (\sigma _{\widehat{n}} \circ \Gamma )_{k,l} \nonumber \\&= \Gamma _{(i+\Delta i) \mathrm {~mod~} n, (j+\Delta j) \mathrm {~mod~} n} ~. \end{aligned}$$

Finally, we view the population either as a configuration $\Gamma$ of spatially positioned $\epsilon$-machines or as a distribution $\textbf{f}$ of $\epsilon$-machine types without regard to spatial location. The fractions $\textbf{f}= \left( \textbf{f}^1, \ldots , \textbf{f}^{15} \right)$ of $\epsilon$-machines of type $T_a$ on $\Gamma$ are $\textbf{f}^a (\Gamma ) = {\Pr }(T_a \in \Gamma )$.

**Fig. 1:**
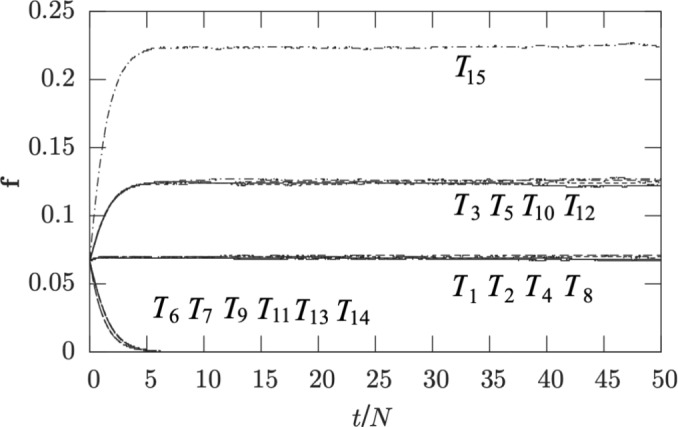
Panmixia population evolution: $\epsilon$-Machine-type distribution $\textbf{f}$ as a function of replication time (*t*/*N*), with $n = 10^3$, $N = 10^6$, $c = 3000$, and $v = 1000$

## Results

### The Panmixia Soup

The least-spatial architecture of $\Gamma$, where $c \approx N$ and $v \approx n$, was previously studied in Crutchfield and Gornerup (2006), and Gornerup and Crutchfield (2008). This is the case of a *panmixia* population, in which all $\epsilon$-machines can interact with any other. When the size of $\Gamma$ is large, $\textbf{f}(t) \equiv \textbf{f}(\Gamma ^t)$ follows a simple population dynamics.

First, for large *N* the discrete-time dynamic is well approximated by continuous time, since each discrete time updates only a single lattice location and this means that changes to $\Gamma ^t$ and so to $\textbf{f}(t)$ are relatively small ($\propto N^{-1})$. Second, the probability of adding an $\epsilon$-machine $T_i$ is that of picking two $\epsilon$-machines $T_a$ and $T_b$, such that $T_a \circ T_b = T_i$, times the probability that the $\epsilon$-machine replaced is not $T_i$. Third, the probability of removing an $\epsilon$-machine $T_i$ is that of picking two $\epsilon$-machines, $T_a$ and $T_b$, such that $T_a \circ T_b \ne T_i$ and also $T_a \circ T_b \ne T_0$, times the probability of picking a $T_i$ to replace. Thus, the rate of change of $\textbf{f}^i (t)$ is given by the differential equation:

8 $$\begin{aligned} \dfrac{d\textbf{f}^i}{dt} = \left( 1 - \textbf{f}^i \right) \sum _{T_a \circ T_b = T_i} \textbf{f}^a \textbf{f}^b - \textbf{f}^i \sum _{\begin{array}{c} T_a \circ T_b \ne T_i \\ T_a \circ T_b \ne T_0 \end{array}} \textbf{f}^a \textbf{f}^b ~, \end{aligned}$$

where the sums run over all pairs satisfying their subscripted condition. That is, $\sum _{T_a \circ T_b = T_i}$ runs over all ordered pairs $(T_a, T_b)$ such that $T_a \circ T_b = T_i$. Equation 8 also determines the steady-state probability distributions of $\epsilon$-machine types, which are simply solutions of:

9 $$\begin{aligned} \dfrac{d\textbf{f}}{dt} = 0 ~. \end{aligned}$$

Figure 1 shows the distribution $\textbf{f}(t)$ over $\epsilon$-machine types as a function of time (replication) for a simulation with $c=3000$ and $v=1000$. This is essentially the same population behavior reported in Crutchfield and Gornerup (2006), and Gornerup and Crutchfield (2008). It was confirmed numerically that Eq. 8 closely predicts the results in Fig. 1. In Fig. 1, all 16 single-state $\epsilon$-machines are shown. They partition themselves into four classes such that those in the same class behave similarly; see Table 1. Six $\epsilon$-machines die away, while nine persist in a closed, self-maintaining, and dynamically stable *meta-machine*, as shown in Crutchfield and Gornerup (2006), and Gornerup and Crutchfield (2008).

**Table 1:** $\epsilon$-Machine-type behavior classes in the panmixia soup of Fig. 1

$\epsilon$-Machine type	Behavior class
$T_{15}$	Fast growth
$T_{3}, T_{5}, T_{10}, T_{12}$	Medium growth
$T_{1}, T_{2}, T_{4}, T_{8}$	No growth
$T_{6}, T_{7}, T_{9}, T_{11}, T_{13}, T_{14}$	Fast decay

### General Replicators

While these $\epsilon$-machine classes can be understood by examining the solutions of Eq. 8 as done in Crutchfield and Gornerup (2006), and Gornerup and Crutchfield (2008), the results can also be directly predicted by examining the algebraic structure of the set $\mathbb {T}$ under the composition operator determined by $\mathbb {M}$. And this observation is critical to predicting the behavior of alternate population architectures. First, we address the panmixia population. The central idea is to find subsets of $\epsilon$-machine types that map onto themselves under the population dynamics; in other words, to identify $\mathbb {M}$-invariant subsets of $\mathbb {T}$.

#### Definition 1

A set *S* of $\epsilon$-machines is a *general replicator* (GR) if for all $a \in \mathbb {T}$ we have:

$$\begin{aligned} \theta (\mathbb {T},a,S) \cup \theta (S, a, \mathbb {T}) \subseteq S \cup \lbrace a \rbrace \end{aligned}$$

and

$$\begin{aligned} \theta (\mathbb {T}, a, S) \cup \theta (S, a, \mathbb {T}) \ne \lbrace a \rbrace ~. \end{aligned}$$

This parallels the definition of an *ideal* in semigroup theory, except that we must be more careful (and less elegant) with the definition since $\theta$ is not a binary relation (Holcombe, 1982).

**Fig. 2:**
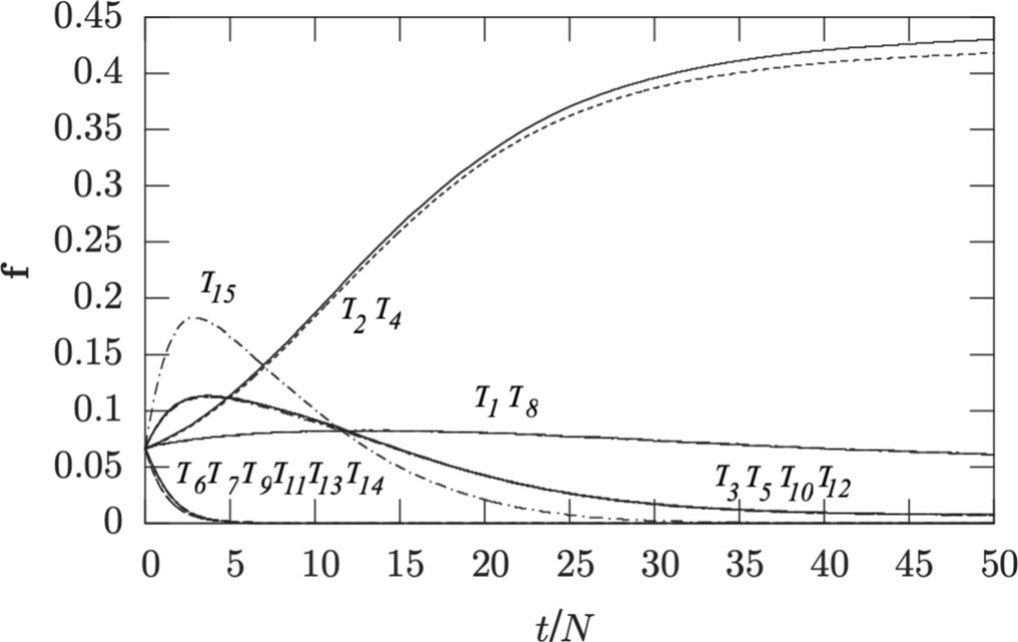
Spatial population dynamics: $\epsilon$-Machine-type distribution $\textbf{f}$ as a function of replication time with $n = 10^3$, $N = 10^6$, and $c=0$

In $\mathbb {T}$ there are many GRs, including $\mathbb {T}$ itself. We intentionally used $\theta$ to exclude the non-$\epsilon$-machine $T_0$ from being considered a GR. By design, $T_0$ cannot be produced. GRs are important because they are the simplest form of replicator. Suppose *S* is a GR. As evolution progresses, elements in the soup are replaced with some element of $\theta (\mathbb {T},a,S)$ or $\theta (S,a,\mathbb {T})$. By the definition of a GR, though, $\theta (\mathbb {T},a,S)$ and $\theta (S,a,\mathbb {T})$ contain elements of *S* and so generally we expect elements of *S* to produce more elements of *S*.

However, not all GRs are equal since some are subsets of others and therefore are produced more readily. There are, in fact, many GRs in $\mathbb {T}$, but there is one GR, call it $\Omega$, that is *minimal* in that no subset of $\Omega$ is a GR, but every GR contains $\Omega$. (Note that, generally, such a set is not guaranteed to exist in a semigroup with a zero element Holcombe, 1982.)

#### Proposition 1

$\Omega = \lbrace T_1, T_2, T_3, T_4, T_5, T_8, T_{10}, T_{12}, T_{15} \rbrace$ is the minimal general replicator in $\mathbb {T}$.

#### Proof

By direct verification of Def. 1 and observing that $\Omega$, short any one of its $\epsilon$-machines, is not a GR.

In the panmixia population, we expect that elements of $\Omega$ come to dominate the soup. And this is what is observed in the simulations (Fig. 1 and Table 1). While Eq. 8 explains the specific values of $\textbf{f}$, an understanding of $\mathbb {M}$’s structure leads to a direct explanation for why the set fast decay is removed from the soup: None of the $\epsilon$-machines in fast decay are in $\Omega$.

### The Spatial Soup

The population that we consider next is that of a spatially configured soup where $c=0$ or $v=0$. In these limits, no spatial mixing is added to the system and the model behaves essentially as an asynchronous, probabilistic cellular automaton. Specifically, each element is replaced by the composition of two of its neighbors if that composition does not result in $T_0$. The pair of neighbors composed is chosen with uniform probability, according to Eq. 4.

Figure 2 shows the $\epsilon$-machine-type distribution $\textbf{f}(t)$ for $c=0$ and $N = 10^6$. Note that initially the population behaves quite similarly to the panmixia case. This is to be expected since the simulations begin with identical initial configurations so at $t=0$ the range of possible interactions is effectively the same for both architectures. As in the panmixia population, elements not in $\Omega$ are quickly removed from the soup.

By $t / N \approx 3.4$, however, the populations start to behave differently. For example, the $\epsilon$-machine $T_{15}$ that is most readily made in the panmixia case is also readily made in the spatial case, until $t/N=3.4$, when it begins to be removed from the population. Again, the $\epsilon$-machines partition themselves into subsets whose elements behave similarly. At early times before $t/N=3.4$, these are identical to those seen in the panmixia population except that $T_2$ and $T_4$ undergo logistic growth, while $T_1$ and $T_8$ do not. Table 2 summarizes the overall behavior, for both early and late times.

**Table 2:** $\epsilon$-Machine-type behavior classes in the two-dimensional spatial population for early and late times for Fig. 2

$\epsilon$-Machine type	Early	Late
$T_{15}$	Fast growth	Decay
$T_{3}, T_{5}, T_{10}, T_{12}$	Medium growth	Decay
$T_{2}, T_{4}$	Logistic growth	Saturation
$T_{1}, T_{8}$	No growth	No growth
$T_{6}, T_{7}, T_{9}, T_{11}, T_{13}, T_{14}$	Fast decay	Removed

Early times: $t/N < 3.4$; late times: $t/N > 3.4$

**Fig. 3:**
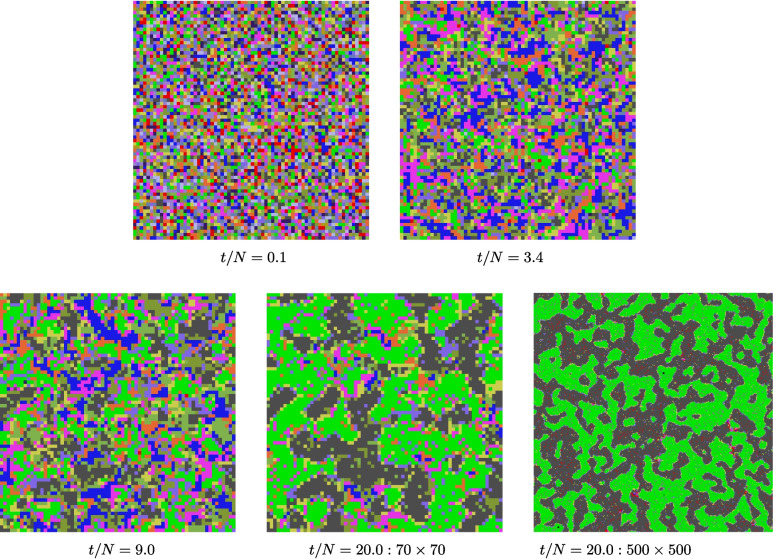
Emergent spatial replicators and their membranes: A $70 \times 70$ region of $\Gamma$ with $n = 10^3$, $N=10^6$, and $c = v = 0$. The last image, however, is of a $500 \times 500$ region. Each color corresponds to one of the 16 $\epsilon$-machine types: $T_2$ is green, $T_4$ is dark gray, $T_1$ is yellow, $T_8$ is purple, and $T_{15}$ is blue

More striking than the evolution of the $\epsilon$-machine-type distribution $\textbf{f}(t)$, however, are the spatial patterns that emerge in $\Gamma ^t$. Figure 3 shows a $70 \times 70$ region of $\Gamma ^t$ at increasing times and one $500 \times 500$ region at a late time. Regions of $T_2$ and $T_4$ form stable domains that grow to dominate the soup. Within a region of $T_2$ or $T_4$ there exist $\epsilon$-machines that cannot be replaced, typically due to an “elastic” collision—an interaction producing the non-$\epsilon$-machine $T_0$ that, by $\theta$, simply leaves the $\epsilon$-machine at the lattice location alone.

When a region of $T_2$ meets a region of $T_4$, $\epsilon$-machines $T_1$ and $T_8$ form on the boundary, since $T_2 \circ T_4 = T_1$ and $T_4 \circ T_2 = T_8$. Moreover, $T_1 \circ T_1 = T_1$ and $T_8 \circ T_8 = T_8$ and so the boundaries are self-sustaining along the interface.

The spatial soup exhibits many of the nontrivial spontaneous patternings common to reaction-diffusion systems that exhibit Turing instability (Turing, 1952; Meinhardt, 1982; Ball, 1999; Hoyle, 2006). Finding a set of reaction-diffusion partial differential equations equivalent to this model and the spatial analog of Eq. 8, however, remains an open problem.

Over a large number of time steps, the spatial population looks essentially like that at $t/N=20.0$ in Fig. 3. If enough time passes, though, the soup eventually divides itself into two connected regions of $T_2$ and of $T_4$, or one will take over completely. This requires an extremely large number of replications. Which $\epsilon$-machine region dominates in the long run appears to be randomly determined. The overall process is highly reminiscent of spinodal decomposition in which a mixed solution separates into stable component phases (Favvas & Mitropoulos, 2008). A more direct connection to the predictions of that theory awaits further effort.

### Spatial-Replicator Domains and Their Membranes

After time $t/N \approx 3.4$, the characters of the spatial and panmixia populations begin to diverge substantially, with patterns emerging in the spatial $\Gamma$. Those patterns consist of *domains* of nearly homogeneous $\epsilon$-machines of type $T_2$ or of type $T_4$. They are separated by domain *walls* that consist of $\epsilon$-machines $T_1$ and $T_8$. As we shall see, these walls play the role of functional membranes that actively translate between the domain $\epsilon$-machines.

**Fig. 4:**
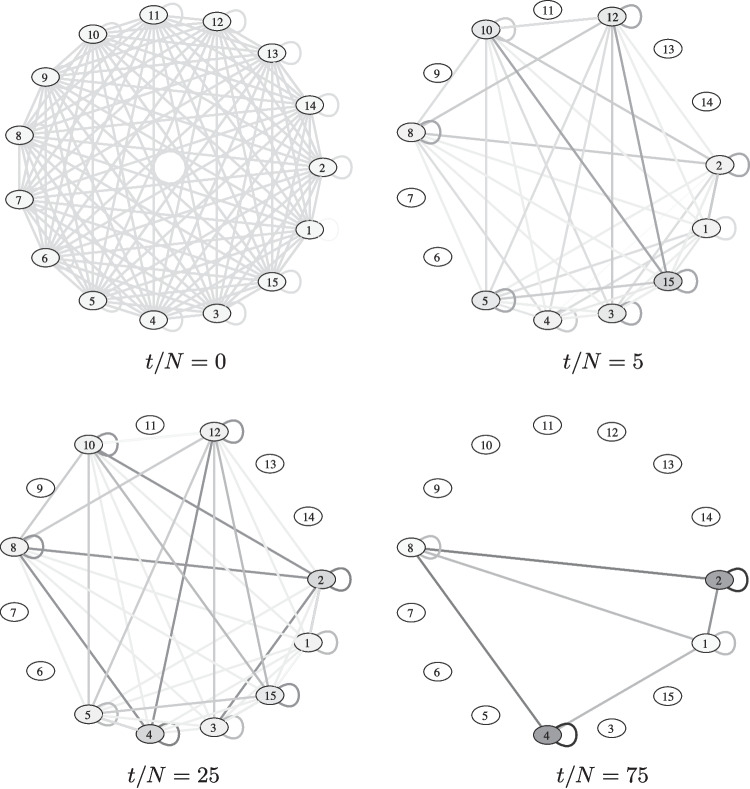
The effective $\epsilon$-machine interactions at various times. The nodes are the 15 possible $\epsilon$-machines. The darkness of their interiors indicates their relative fractions $\textbf{f}^i$ in the populations. The edges connecting them indicate the frequency of their interactions; darker is more frequent

To summarize, then, the first observation about the spatial population is that it is completely differently organized. The persistent set of $\epsilon$-machines differs markedly from those found with panmixia, as do their roles and interactions. Why did they emerge when the population is embedded in space? We can explain the emergence of these structures by defining a mechanism in the interaction network $\mathbb {M}$ that affects the population dynamics when there is sufficient “spatial memory”. This mechanism is called a *spatial replicator* (SR).

#### Definition 2

A set *S* of $\epsilon$-machines is a *spatial replicator* (SR) if

$$\begin{aligned} \theta (S,S,\theta (S,S,\mathbb {T}))&\subseteq S,\\ \theta (S,S,\theta (\mathbb {T},S,S))&\subseteq S,\\ \theta (\theta (S,S,\mathbb {T}),S,S)&\subseteq S, \textrm{ and} \\ \theta (\theta (\mathbb {T},S,S),S,S)&\subseteq S. \end{aligned}$$

Notice that this is similar to the definition of a GR, except that SRs require two applications of $\theta$ to produce an element of *S*. Due to this, SRs only replicate when there is spatial information storage: If *S* is a SR, an element of *S* must compose with an $\epsilon$-machine in $\mathbb {T}$ to produce an $\epsilon$-machine $T_y$. $T_y$ must then compose with an element of *S* again to produce a new element of *S*.

In the panmixia population, there is no notion of adjacency. Therefore, $T_y$ does not have an increased chance of interacting with an element of *S* again. However, space allows a way for SRs to replicate: If by chance a sufficiently large domain of $\Gamma$ consists only of elements of some SR *S*, $\epsilon$-machines on the domain border will be replaced by elements of $\theta (S, S, \mathbb {T})$ or $\theta (\mathbb {T}, S, S)$. Later, these boundary $\epsilon$-machines will be replaced with elements of

10 $$\begin{aligned} \theta (S,S,&\theta (S,S,\mathbb {T})) \cup \theta (S,S,\theta (\mathbb {T},S,S)) \nonumber \\&\cup \theta (\theta (S,S,\mathbb {T}),S,S) \cup \theta (\theta (\mathbb {T},S,S),S,S) ~, \end{aligned}$$

which are all elements of *S*. Thus, a domain of *S* will grow. This argument lays out how the algebraic structure that space adds to $\mathbb {M}$ leads to domain growth. In addition, since $\theta (S, S, S) = S$, domains of *S* are self-maintaining.

As with a GR, one expects that if $S'$ is a SR and is also a subset of some other SR *S*, then $S'$ eventually will come to dominate the soup. For the spatial population of single-state $\epsilon$-machines, it is easy to check that $\{ T_1, T_2, T_4, T_8 \}$ is a SR. Dynamically, the membranes of $T_1$ and $T_8$ grow their respective domains of $T_4$ and $T_2$. Unsurprisingly, then, $T_2$ and $T_4$ come to dominate $\Gamma$. In contrast, $T_{15}$—an $\epsilon$-machine made so readily by the panmixia population—begins to dominate the simulation until “spatial memory” begins to act. Beyond that point $T_{15}$ is replaced by SRs.

**Fig. 5:**
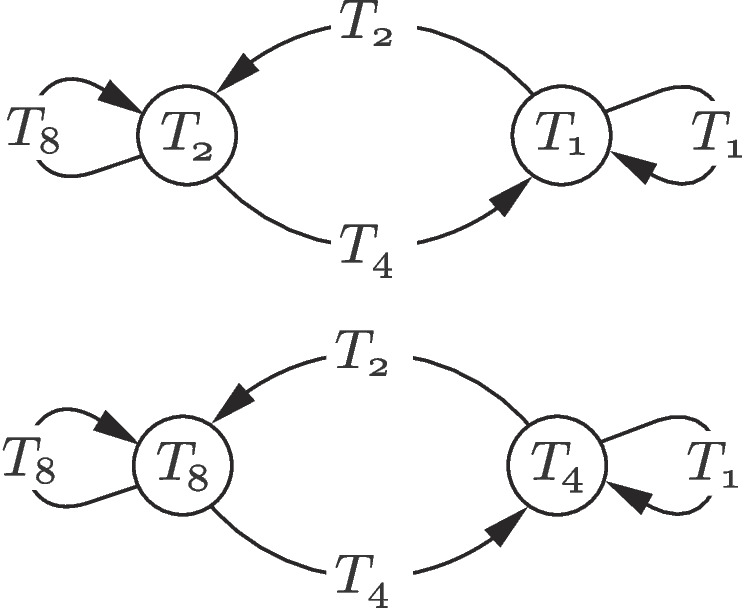
The $\epsilon$-machine interaction network at long times in the spatial soup. An arrow labeled $T_b$ going from node $T_a$ to node $T_c$ denotes $T_c = T_b \circ T_a$

Figure 4 shows how these interactions develop over time by giving the effective $\epsilon$-machine interactions on an undirected graph, where the darkness of the $\epsilon$-machine nodes indicates their relative frequencies $\textbf{f}^i$ and the darkness of the connecting edges indicates the relative frequency of interactions. As a complement to this, Fig. 5 gives the $\epsilon$-machine interaction network at long times. This should be compared to the nine-$\epsilon$-machine meta-machine in the panmixia soup: see Fig. 3 of Crutchfield and Gornerup (2006), and Gornerup and Crutchfield (2008).

The explanation for why a spatial population organizes differently and, in effect, selects different individuals for different roles relies on SRs, a local mechanism. The domains and walls, however, are nonlocal structures. Given a local dynamic that produces them, how do we describe their global structure?

To do this, we adapt the computational mechanics analysis of emergent domains and particles in deterministic, synchronous cellular automata (CAs) (Crutchfield & Hanson, 1993; Hanson & Crutchfield, 1992, 1997) to the stochastic, asynchronous spatial population dynamics here. Speaking informally, a domain is a patch of spacetime that has the same “texture” when shifted in time, space, or both. How much one must shift each domain so that its texture maps onto itself is one crude measure of the structural complexity of the domain.

#### Definition 3

A *domain* is a set of configurations $\Gamma ^{S} = \{ \Gamma _{i,j} \in S \}$, where *S* is an SR, that satisfies:

1. Temporal-shift invariance: $\Gamma ^{S} = \Phi \Gamma ^{S}$, and
2. Spatial-shift invariance: $\Gamma ^{S} = \sigma ^{\widehat{n}} \Gamma ^{S}$ for some spatial offset $\widehat{n}$.

It is straightforward to see that, in the spatial population, homogeneous regions of SRs—$\{T_2\}$ and $\{T_4\}$—are domains. Determining which additional $\epsilon$-machines—the nonreplaceable ones mentioned above—can be embedded in these domains, such that the regions are still domains, is a more difficult calculation that we will not attempt here.

The membranes separating the domains are complementary $\epsilon$-machine types that actively function to translate between domains on either side, being composable with the $\epsilon$-machine types in those domains. And, over time, sets of membrane $\epsilon$-machines map back into themselves. In the spatial population, as noted above (Fig. 5), $T_1 = T_2 \circ T_4$ and $T_8 = T_4 \circ T_2$. The definition of a membrane replicator and a membrane express these formally.

#### Definition 4

A set *M* of $\epsilon$-machines is a *membrane replicator* (MR) if there are two domain SRs, *S* and $S^\prime$, such that

$$\begin{aligned} \theta (S,M,\theta (S,M,S^\prime ))&\subseteq M,\\ \theta (S,M,\theta (S^\prime ,M,S))&\subseteq M,\\ \theta (\theta (S,M,S^\prime ),M,S^\prime )&\subseteq M, \textrm{ and} \\ \theta (\theta (S^\prime ,M,S),M,S^\prime )&\subseteq M. \end{aligned}$$

Notice that this is similar to the definition of an SR, except that MRs require the bounding domain SRs to produce $\epsilon$-machines in the MR.

#### Definition 5

A *membrane* between two domain SRs *S* and $S^\prime$ is a set of configurations:

11 $$\begin{aligned} \Gamma ^{SS^\prime } = \Gamma ^{S} M \Gamma ^{S^\prime } ~, \end{aligned}$$

where *M* is a membrane replicator, that is temporal-shift invariant:

12 $$\begin{aligned} \Gamma ^{SS^\prime } = \Phi \Gamma ^{SS^\prime } ~. \end{aligned}$$

To emphasize, $\Gamma$ is the population. $\Gamma ^S$ is the population restricted to $\epsilon$-machine subset *S* in a domain. $\Gamma ^{SS^\prime }$ is the population restricted to those individuals in the membrane between the domain of *S* and domain $S^\prime$.

Observe that membranes, like domains, are temporally invariant, but, unlike domains, they are *not* spatially shift invariant. This is what it means, in fact, for a structure to have a location in space. In terms of emergent structures domains are local in the sense that they can be characterized by describing a small spatial region. Whereas, membranes bound entire regions and therefore require a chain of nonlocal properties (interactions, e.g.) to describe.

**Fig. 6:**
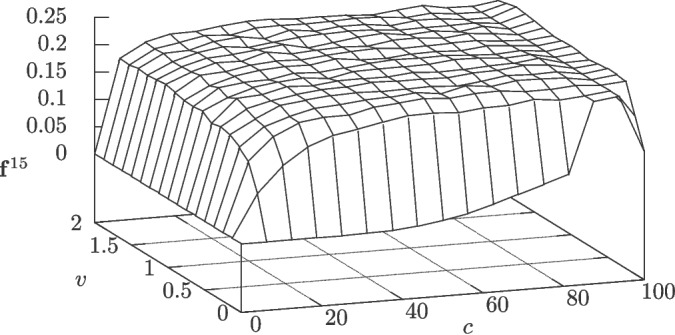
$\textbf{f}^{15} (t)$ after $t/N = 50$ time steps as a function of *c* and *v* with $N=10^6$

**Fig. 7:**
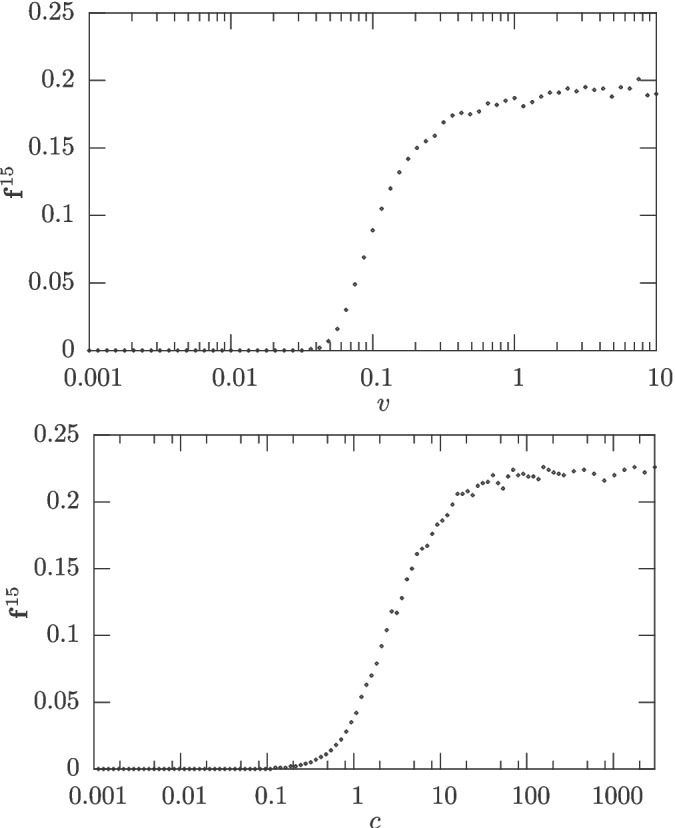
Top panel: $\textbf{f}^{15} (t)$ after $t/N = 50$ time steps with $N=10^6$ and $c=10.0$. Bottom panel: $\textbf{f}^{15}$ after $t/N = 50$ time steps with $N=10^6$ and $v=1.0$. Note that in both, the horizontal axis is logarithmic

Membranes translate between the, possibly distinct, kinds of information stored in the bounded SR domains. This view parallels that of, for example, the particles which perform computations in evolved cellular automata (Crutchfield & Mitchell, 1995). Specifically, when the spatial population dynamic is deterministic, then SR domains and membranes are directly analogous to the domains and particles of cellular automata computational mechanics (Crutchfield & Hanson, 1993; Hanson & Crutchfield, 1992, 1997). The particular class of population dynamical systems considered here differ, however, in important ways: They have an asynchronous, stochastic spatial update. A more detailed framing of these differences and the methods to analyze them will be reported elsewhere.

In summary, Defs. 3, 4, and 5 specify algebraic constraints that, in principle, can be solved to find SR domains and membranes. Even in the simpler case of the computational mechanics analysis of deterministic CAs, solving the analogous set of constraints, though well defined, is at present difficult. And so, we will leave detailing the algorithms for these calculations to a sequel.

### Intermediate Spatial Populations

The $\epsilon$-machine soups here were specifically designed to permit investigation of populations that are neither completely spatial nor completely nonspatial by varying the mixing parameters *c* and *v*. To avoid presenting extremely high-dimensional data, we pick a metric that distinguishes the spatial versus nonspatial population dynamics and observe how that metric varies with *c* and *v*. For the metric we use $\textbf{f}^{15} (t)$, the percentage of $\epsilon$-machines of type $T_{15}$ in $\Gamma ^t$. In the nonspatial simulation, $\textbf{f}^{15} \approx 0.223$ after $t/N = 50$ steps, while $\textbf{f}^{15} \approx 0.0$ after $t/N = 50$ in the spatial model. In this way, Fig. 6 shows the transition between spatial and nonspatial behavior for the model as a function of *c* and *v*.

The two plots in Fig. 7 provide a clearer demarcation of the transition at which spatial memory is lost by showing slices along the *v* and *c* axes. Notably, the plots show that the “effect of space” is logistic in $\log c$ or $\log v$. This indicates that populations are sensitive to both parameters in similar ways, even though the physical realizations of varying *c* (number of $\epsilon$-machines swapped) and *v* (spatial range of mixing) are quite different.

## Discussion

The analysis demonstrated that the capacity for using spatial memory can be determined by examining the interaction network. Moreover, it also showed that the behavior of populations which are neither completely spatial nor completely nonspatial exhibit a systematic dependence on the degree of mixing. One can imagine several ways to generalize the definition of SRs—as the definition presented above is sufficient, but by no means necessary—for the spatial populations to behave differently from the nonspatial populations.

While the exact population dynamics of $\textbf{f}$ may be captured by an ODE such as Eq. 8, this coarse-graining is not useful to understanding the population dynamics’ key structural properties—the pattern formation highlighted in the spatial case. Structural analysis of the interaction network, focusing specifically on its algebraic properties, was essential to understanding the mechanisms that drive the spontaneous emergence of organization. To this end, we drew a useful, if preliminary, connection to the computational mechanics analysis of domains and walls structures in spatially extended systems.

The membranes observed (and defined) here are not merely concentration gradients, as seen in familiar pattern formation systems. Rather, they are entities that, due to their interaction specificity, actively translate between replicators in neighboring domains. The resulting spatial organization suggests that one gets spontaneous compartmentalization without designing it in at the beginning. Recall that compartmentalization is often cited as one of the key early evolutionary steps on the road to increasing biological complexity (Maynard Smith & Szathmary, 1995). It is not such a difficult step, after all. The $\epsilon$-machine soup is a very simple system, built with a minimal set of physical, chemical, and biological assumptions. The emergence of GRs, SRs, and MRs as metamachines highlights rich diversity of distinct levels of population complexity.

It is perhaps no surprise if we mention that analogous investigations of one-dimension $\epsilon$-machine soups leads to similar results. This and the two-dimensional, intermediate dimensional, and effectively infinite dimensional (panmixia) soups lead one to wonder about the role of the three dimensions in which we know that life arose. Is there something special?

Constructively, in concert with natural spontaneous pattern formation, evolution may very well commandeer structures of different spatial dimension as ways that insure various kinds of functionality or intrinsic computation. We appreciate that evolution is opportunistic and takes advantage of nature’s propensity to spontaneously organize. And so, is three-dimensional space more advantageous in, say, the range of structures that it supports, compared to one and two dimensions? Or does evolution take advantage of all possible dimensions? The empirical evidence speaks rather clearly, if not to the former question, then to the latter: genetic information is stored in one-dimensional structures, biological cells sport two-dimensional membranes, and organisms move and behave in three dimensions.

## Data Availability

No datasets were generated or analyzed during the current study.

## References

[CR1] Ackermann, J., Kirner, T., & Klapp, S. H. L. (2006). Complex pattern formation of simple biochemical amplification reactions in micro-structured flow reactors. *Zeitschrift fur Naturforschung A,**61*(1–2), 60–68.

[CR2] Adami, C., & Brown, C. T. (1994). Evolutionary learning in the 2d artificial life system ‘Avida’. In *Artificial Life 4* (pp. 377–381). MIT Press.

[CR3] Ball, P. (1999). *The self-made tapestry: Pattern formation in nature*. New York: Oxford University Press.

[CR4] Barnett, N., & Crutchfield, J. P. (2015). Computational mechanics of input-output processes: Structured transformations and the -transducer. *Journal of Statistical Physics, 161*(2), 404–451.

[CR5] Castilian, R. F., Cech, T. R., & Atkins, J. F. (Eds.). (1999). *The RNA world*. Cold Spring Harbor, Massachusetts: Cold Spring Harbor Laboratory Press.

[CR6] Crutchfield, J. P. (1994). The calculi of emergence: Computation, dynamics, and induction. *Physica D,**75*, 11–54.

[CR7] Crutchfield, J. P., & Gornerup, O. (2006). Objects that make objects: The population dynamics of structural complexity. *Journal of The Royal Society Interface,**3*, 345–349. 16849243 10.1098/rsif.2006.0114 PMC1578741

[CR8] Crutchfield, J. P., & Hanson, J. E. (1993). Turbulent pattern bases for cellular automata. *Physica D,**69*, 279–301.

[CR9] Crutchfield, J. P., & Mitchell, M. (1995). The evolution of emergent computation. *Proceedings of the National Academy of Sciences,**92*, 10742–10746. 10.1073/pnas.92.23.10742 PMC40688 11607588

[CR10] Crutchfield, J. P., & Shalizi, C. R. (1999). Thermodynamic depth of causal states: Objective complexity via minimal representations. *Physical Review E,**59*(1), 275–283.

[CR11] Crutchfield, J. P., & Young, K. (1989). Inferring statistical complexity. *Phys Revista de Letras,**63*, 105–108. 10.1103/PhysRevLett.63.105 10040781

[CR12] Eigen, M. (1971). Self-organization of matter and the evolution of biological macromolecules. *Naturwissenschaften,**58*, 465–523. 4942363 10.1007/BF00623322

[CR13] Eigen, M., & Schuster, P. (1977). The hypercycle. A principle of natural self-organization. Part A: Emergence of the hypercycle. *Naturwissenschaften,**64*, 541–565. 593400 10.1007/BF00450633

[CR14] Ellison, C. J., Mahoney, J. R., & Crutchfield, J. P. (2009). Prediction, retrodiction, and the amount of information stored in the present. *Journal of Statistical Physics,**136*(6), 1005–1034.

[CR15] Favvas, E. P., & Mitropoulos, ACh. (2008). What is spinodal decomposition? *Journal of Engineering Science and Technology Review,**1*, 25–27.

[CR16] Fontana, W. (1991). Algorithmic chemistry. In C. Langton, C. Taylor, J. D. Farmer, & S. Rasmussen (Eds.), *Artificial Life II, volume XI of Santa Fe Institute Studies in the Sciences of Complexity* (pp. 159–209). Redwood City, California: Addison-Wesley.

[CR17] Gornerup, O., & Crutchfield, J. P. (2008). Hierarchical self-organization in the finitary process soup. *Artificial Life Journal,**14*(3), 245–254. 10.1162/artl.2008.14.3.14301 18489247

[CR18] Hanson, J. E., & Crutchfield, J. P. (1992). The attractor-basin portrait of a cellular automaton. *Journal of Statistical Physics,**66*, 1415–1462.

[CR19] Hanson, J. E., & Crutchfield, J. P. (1997). Computational mechanics of cellular automata: An example. *Physica D,**103*, 169–189.

[CR20] Holcombe, W. M. L. (1982). *Algebraic automata theory*. Cambridge: UK, Cambridge University Press.

[CR21] Hoyle, R. (2006). *Pattern formation: An introduction to methods*. New York: Cambridge University Press.

[CR22] Kirner, T., Ackermann, J., Steen, D., Ehricht, R., Ellinger, T., Foerster, P., & McCaskill, J. S. (2000). Complex patterns in a trans-cooperatively coupled DNA amplification system. *Chemical Engineering Science,**55*, 245–256.

[CR23] Kirner, T., Steen, D., McCaskill, J. S., & Ackermann, J. (2002). Biochemical amplification waves in a one-dimensional microflow system. *Journal of Physical Chemistry B,**106*(17), 4525–4532.

[CR24] Marzen, S. E., & Crutchfield, J. P. (2018). Optimized bacteria are environmental prediction engines. *Physical Review E,**98*, 012408. 30110764 10.1103/PhysRevE.98.012408

[CR25] Marzen, S. E., & Crutchfield, J. P. (2025). Prediction and dissipation in molecular sensors: Conditionally Markovian channels driven by memoryful environments. *Bulletin of Mathematical Biology,**82*, 1–46. 10.1007/s11538-020-00694-2 31993762

[CR26] Maynard Smith, J., & Szathmary, E. (1995). *The major transitions in evolution*. Oxford: W. H. Freeman/Spektrum.

[CR27] Meinhardt, H. (1982). *Models of biological pattern formation*. London: Academic Press.

[CR28] Rasmussen, S., Knudsen, C., & Feldberg, R. (1992). Dynamics of programmable matter. In *Artificial Life II: Proceedings of an interdisciplinary workshop on the synthesis and simulation of living systems (Santa Fe institute studies in the sciences of complexity, Vol. 10)*. Addison-Wesley.

[CR29] Ray, T. S. (1991). An approach to the synthesis of life. In C. Langton, C. Taylor, J. D. Farmer, & S. Rasmussen (Eds.), *Artificial Life II, volume XI of Santa Fe institute studies in the sciences of complexity* (pp. 371–408). Redwood City, California: Addison-Wesley.

[CR30] Samáry, E., & Demeter, L. (1987). Group selection of early replicators and the origin of life. *Journal of Theoretical Biology,**128*, 463–486. 2451771 10.1016/s0022-5193(87)80191-1

[CR31] Turing, A. M. (1952). The chemical basis of morphogenesis. *Transactions of the Royal Society B,**237*, 5.

